# The association between social jetlag and poor health and its (nutritional) mechanisms

**DOI:** 10.1017/S1368980022001161

**Published:** 2022-09

**Authors:** Emma J Bouman, Femke Rutters

**Affiliations:** 1 Amsterdam UMC location Vrije Universiteit Amsterdam, Epidemiology and Data Science, De Boelelaan 1117, Amsterdam, Netherlands; 2 Amsterdam Public Health, Health Behaviors & Chronic Diseases, Amsterdam, The Netherlands

In the most recent issue of Public Health Nutrition 2022, Al Khatib *et al*. published an interesting paper on the associations and interactions between sleep duration and social jetlag with nutrient intake and cardio-metabolic risk factors in a nationally representative UK population. They showed in a group of ∼5000 adults (19–64 years) that short sleep and irregular sleep timing were associated with higher intakes of non-milk extrinsic sugars, and that irregular sleep timing was associated with lower fibre intake in those with adequate sleep duration^(1)^.

The majority of the paper focuses on the duration of sleep, but the authors also showed irregular sleep timing to be associated with poorer nutrient intake. Specifically, they focus on irregular sleep timing measured as social jetlag. This relatively new construct of social jetlag is defined as the discrepancy between work schedules, social obligations and biological needs^(2)^. Especially people with a late chronotype (night owls) have social jetlag. Night owls go to sleep late during workdays (their natural preference), but have to get up early due to work obligations. During the weekend, they switch to their natural rhythm of going to bed late and getting up late, trying to catch up on the sleep debt build up during the week by increasing sleep duration during the weekend. This causes chronic variability in sleep timing, namely every week, which we call social jetlag.

Social jetlag is operationalised as the difference in midpoint of sleep between work and weekend/free days^(3)^. For example, when during the week, a person goes to bed at 11 PM and wakes up at 7 AM, the midpoint of sleep is 3 AM. During the weekend he/she goes to bed at 1 AM and wakes up at 9 AM, the midpoint of sleep being 5 AM. The difference in midpoints of sleep is 2 h, which means this person has a social jetlag of 2 h. Most studies to date report social jetlag using cut-off scores, with no social jetlag being less than 1 h of difference in midpoints of sleep, moderate being 1–2 h and high social jetlag being ≥ 2 h difference. Social jetlag prevalence changes with age, with 19 % of the teenagers in the Netherlands reporting no social jetlag^(4)^, compared to 61 % in the elderly population^(5)^. Overall, despite age differences, these studies show that social jetlag is highly prevalent in our current 24-h society.

But is it a bad thing to have social jetlag and to have this reoccurring high variability in sleep timing? Systematic reviews by Beauvalet *et al*.^(6)^, Sun *et al*.^(7)^ and Caliandro *et al*.^(8)^ showed that social jetlag has negative consequences for health, including poorer psychological health as well as poorer cardiometabolic risk profiles, for example, higher prevalence of obesity. There are several hypotheses on the mechanisms explaining these negative effects. First, we have to explain that social jetlag causes circadian misalignment. The circadian clock governs virtually all processes in the human body, including sleep–wake behaviour. Circadian misalignment describes the off-set between sleep–wake cycles and clock-regulated physiology^(9)^. By sleeping at irregular times, social jetlag disturbs the circadian clock and the processes it regulates. For example, by causing circadian misalignment, social jetlag disrupts the hypothalamic–pituitary–adrenal axis, which in turn promotes metabolic and glycaemic changes^(10,11)^.

Other possible explaining mechanisms include behavioural changes. These are for example unhealthy food choices, as described in the paper by Al Khatib *et al*. (high non-milk extrinsic sugars and low fibre intake), but also by others^(12–15)^. Additionally, social jetlag is associated with high caffeine intake in teenagers^(16)^. In addition to poorer food choices, social jetlag is thought to increase sedentary behaviours as shown in our own small cross-sectional study, in which those with social jetlag had lower physical activity levels^(17)^, confirmed by Alves *et al*.^(18)^ Moreover, in addition to decreasing physical activity, an increase in other sedentary behaviours, such as gaming was observed^(19)^. When present on the long-term these negative lifestyle behaviours can cause a positive energy balance, which, when chronic, leads to an increase in waist circumference and BMI, and higher risk of obesity. This in turn can lead to poorer glycaemic control and increased HbA1c^(20)^.

Finally, concomitant disorders such as depression, fatigue or other sleep disorders could also promote increases in BMI and HbA1c via changes in metabolic and glycaemic control^(21)^. However, as many of the studies were observational and predominantly cross-sectional, further research is required to explore the underlying mechanisms of the negative effects of social jetlag. For now, research to date suggests that social jetlag is common (>50 % of the population has it) and is associated with negative effects on health. Health care providers should be more aware of the magnitude and impact of social jetlag. Efforts towards educating people about the importance of sleep and regular sleep timing could be a first strategy to help improve health.

## References

[1] Al Khatib H , Dikariyanto V , Bermingham KM et al. (2022) Short sleep and social jetlag are associated with higher intakes of non-milk extrinsic sugars, and social jetlag is associated with lower fibre intakes in those with adequate sleep duration: A cross-sectional analysis from the national diet and nutrition survey rolling programme (years 1–9). Public Health Nutr 18, 1–12. 10.1017/S1368980022000167 PMC9991673 35039109

[2] Roenneberg T , Allebrandt KF , Merrow M et al. (2012) Social jetlag and obesity. Curr Biol 22, 939–943. 22578422 10.1016/j.cub.2012.03.038

[3] Wittmann M , Dinich JF , Merrow M et al. (2006) Social jetlag: misalignment of biological and social time. Chronobiol Int 23, 497–509. 16687322 10.1080/07420520500545979

[4] Kocevska DA-O , Lysen TA-OX , Dotinga A et al. (2021) Sleep characteristics across the lifespan in 1.1 million people from the netherlands, United Kingdom and United States: A systematic review and meta-analysis. Nat Hum Behav 5, 113–122. 33199855 10.1038/s41562-020-00965-x

[5] Koopman ADM , Rauh SP , van’t Riet E et al. (2017) The association between social jetlag, the metabolic syndrome, and type 2 diabetes mellitus in the general population: the new hoorn study. J Biol Rhythms 32, 359–368. 28631524 10.1177/0748730417713572 PMC5564947

[6] Beauvalet JC , Quiles C , Oliveira MAB et al. (2017) Social jetlag in health and behavioral research: a systematic review. ChronoPhysiol Ther 7, 19–31.

[7] Sun W , Ling J , Zhu X et al. (2019) Associations of weekday-to-weekend sleep differences with academic performance and health-related outcomes in school-age children and youths. Sleep Med Rev 46, 27–53. 31060028 10.1016/j.smrv.2019.04.003

[8] Caliandro R , Streng AA , van Kerkhof LWM et al. (2021) Social jetlag and related risks for human health: a timely review. Nutrients 13, 4543. 34960096 10.3390/nu13124543 PMC8707256

[9] Baron KG & Reid KJ (2014) Circadian misalignment and health. Int Rev Psychiatry 26, 139–154. 24892891 10.3109/09540261.2014.911149 PMC4677771

[10] Nader N , Chrousos GP & Kino T (2010) Interactions of the circadian CLOCK system and the HPA axis. Trends Endocrinol Metab 21, 277–286. 20106676 10.1016/j.tem.2009.12.011 PMC2862789

[11] Nieuwenhuizen AG & Rutters F (2008) The hypothalamic-pituitary-adrenal-axis in the regulation of energy balance. Physiol Behav 94, 169–177. 18275977 10.1016/j.physbeh.2007.12.011

[12] Silva CM , Mota MC , Miranda MT et al. (2016) Chronotype, social jetlag and sleep debt are associated with dietary intake among Brazilian undergraduate students. Chronobiol Int 33, 740–748. 27070173 10.3109/07420528.2016.1167712

[13] Suikki T , Maukonen M , Partonen T et al. (2021) Association between social jet lag, quality of diet and obesity by diurnal preference in Finnish adult population. Chronobiol Int 38, 720–731. 33557623 10.1080/07420528.2021.1876721

[14] Mota MC , Silva CM , Balieiro LCT et al. (2019) Association between social jetlag food consumption and meal times in patients with obesity-related chronic diseases. PLoS ONE 14, e0212126. 30753224 10.1371/journal.pone.0212126 PMC6372231

[15] Yoshizaki T & Togo F (2021) Objectively measured chronotype and social jetlag are associated with habitual dietary intake in undergraduate students. Nutr Res 90, 36–45. 34038836 10.1016/j.nutres.2021.04.003

[16] Mathew GA-O , Reichenberger DA , Master L et al. (2021) Too jittery to sleep? Temporal associations of actigraphic sleep and caffeine in adolescents. Nutrients 14, 31. 35010906 10.3390/nu14010031 PMC8746933

[17] Rutters F , Lemmens SG , Adam TC et al. (2014) Is social jetlag associated with an adverse endocrine, behavioral, and cardiovascular risk profile? J Biol Rhythms 29, 377–383. 25252710 10.1177/0748730414550199

[18] Alves MS , Andrade RZ , Silva GC et al. (2017) Social jetlag among night workers is negatively associated with the frequency of moderate or vigorous physical activity and with energy expenditure related to physical activity. J Biol Rhythms 31, 83–93. 10.1177/0748730416682110 28006966

[19] Hamre R , Smith ORF , Samdal O et al. (2022) Gaming behaviors and the association with sleep duration, social jetlag, and difficulties falling asleep among norwegian adolescents. Int J Environ Res Public Health 19, 1765. 35162788 10.3390/ijerph19031765 PMC8834670

[20] Kahn SE , Hull RF , Utzschneider KM et al. (2006) Mechanisms linking obesity to insulin resistance and type 2 diabetes. Nature 444, 840–846. 17167471 10.1038/nature05482

[21] Fárková E , Šmotek M , Bendová Z et al. (2021) Chronotype and social jet-lag in relation to body weight, appetite, sleep quality and fatigue. Biol Rhythm Res 52, 1205–1216.

